# Experimental Induction of Tenacibaculosis in Atlantic Salmon (*Salmo salar* L.) Using *Tenacibaculum maritimum*, *T. dicentrarchi*, and *T. finnmarkense*

**DOI:** 10.3390/pathogens10111439

**Published:** 2021-11-05

**Authors:** Joseph P. Nowlan, Scott R. Britney, John S. Lumsden, Spencer Russell

**Affiliations:** 1 Center for Innovation in Fish Health, Vancouver Island University, Nanaimo, BC V9R 5S5, Canada; Scott.Britney@viu.ca (S.R.B.); Spencer.Russell@viu.ca (S.R.); 2 Department of Pathobiology, University of Guelph, Guelph, ON N1G 2W1, Canada; jsl@uoguelph.ca

**Keywords:** aquaculture, tenacibaculosis, mouthrot, bath-exposure, dysbiosis, histopathology, qPCR

## Abstract

There is a limited understanding of the pathogenesis of tenacibaculosis in Atlantic salmon (*Salmo salar* L.) and there are few reproducible exposure models for comparison. Atlantic salmon were exposed via bath to *Tenacibaculum maritimum*, *T. dicentrarchi*, or *T. finnmarkense,* and were then grouped with naïve cohabitants. Mortalities had exaggerated clinical signs of mouthrot, a presentation of tenacibaculosis characterized by epidermal ulceration and yellow plaques, on the mouth and less frequently on other tissues. Histopathology showed tissue spongiosis, erosion, ulceration, and necrosis ranging from mild to marked, locally to regionally extensive with mats of intralesional bacteria on the rostrum, vomer, gill rakers, gill filaments, and body surface. Exposure to *T. maritimum* resulted in less than a 0.4 probability of survival for both exposed and cohabitants until Day 21. Exposures to *T. dicentrarchi* resulted in 0 and 0.55 (exposed), and 0.8 and 0.9 (cohabitant) probability of survival to Day 12 post-exposure, while *T. finnmarkense* had a 0.9 probability of survival to Day 12 for all groups. This experimental infection model will be useful to further investigate the pathogenesis of tenacibaculosis, its treatment, and immunity to *Tenacibaculum* species.

## 1. Introduction

Several *Tenacibaculum* bacterial species are putative pathogens associated with te- nacibaculosis in fishes [1,2,3,4,5,6]. In British Columbia (Canada; BC), *Tenacibaculum* species are commonly isolated from Atlantic salmon (*Salmo salar* L.) with mouthrot; a regional, clinical presentation of tenacibaculosis, often characterized by epidermal ulcerations and development of yellow plaques around the mouth [1,5,6,7,8]. 

Previous research exposing Atlantic salmon smolts to BC isolates of *T. maritimum* described epidermal ulceration and bacterial plaques on the mouth and less frequently on other tissues [6]; wherein exposure to *T. maritimum* (isolate TmarCan15-1, 16-1, 16-5) at 10^5^–10^7^ cells mL^−1^ induced >84% and >27% mortality of the exposed and cohabitant population [6]. Atlantic salmon infected with *T. maritimum* TmarCan16-1 predominately had ulcerations of the perioral epidermis and dissemination of the bacteria into the tooth pulp, and the authors proposed this as a potential route for the development of systemic infections [5,6]. *T. dicentrarchi* TdChD05 and 35/09^T^, and *T. finnmarkense* HFJ^T^ and Tsp.2 have likewise been used in exposure models [4,9]. Bath immersion with 10^5^–10^6^ colony-forming-units/cells mL^−1^ resulted with 65% (TdChD05 [4]), 20% (35/09^T^ [4]), 80% (HFJ^T^ [9]) and 10% (Tsp.2 [9]) cumulative mortality, respectively, in exposed Atlantic salmon [4,9]. Unlike a previous report [6], where mainly perioral lesions were noted, other studies recorded the presence of ulcerations at multiple locations on the fish [4,9]. These results indicate that *Tenacibaculum* spp. may be able to establish and induce disease in multiple tissues. Variation in tissue tropisms could be related to *Tenacibaculum* species being opportunistic pathogens, where dysbiosis [10,11], variation in isolate virulence, and host genetics may facilitate tenacibaculosis. More work is needed to support how tenacibaculosis is established and how the bacteria become systemic, as well as understanding the mechanisms for mortality in Atlantic salmon smolts.

Tenacibaculosis at BC aquaculture sites is treated through antibiotic application, primarily florfenicol; however, sulfadimethoxine and ormetoprim are also used. As of 2021, only one commercial vaccine for *T. maritimum* is available in Spain for use in turbot (*Psetta maxima* L.) (ICTHIOVAC^®^-TM, Hipra Laboratories) [12]. Vaccines for other aquaculture fishes (e.g., Atlantic salmon) are warranted as *Tenacibaculum* outbreaks lead to direct profit loss associated with fish mortality in addition to the associated cost of serial applications of antibiotics [13]. Before new vaccines can be developed, repeatable experimental infection models are required in target species such as Atlantic salmon.

This work aims to create a repeatable experimental infection model in Atlantic salmon by exposure to isolates most similar to *T. maritimum* NLF-15 (*Tmar*), *T. dicentrarchi* TdChD04 (*Tdic*), or *T. finnmarkense* Tsp.2 (*Tfinn*), and tracking fish pathology and mortality over time. The development of a repeatable infection model will be beneficial for vaccine testing as well as to help understand the pathogenesis of mouthrot in BC.

## 2. Results

### 2.1. Bacterial Isolate Collection

Throughout the experiment, 41 isolates were collected from deceased or moribund fish. Twenty-four isolates were from *Tdic* exposed fish, 1 isolate was from *Tfinn* exposed fish, 12 isolates were from *Tmar* exposed fish and 4 isolates were from *Tmar* cohabitant fish. Isolates were yellow-pigmented, Gram-negative, and were elongated bacilli to filamentous. qPCR testing on 41 collected isolates indicated that all isolates from the *Tdic* exposure were *Tdic*, the isolate from the *Tfinn* exposure was *Tdic*, three isolates from the *Tmar* exposure were *Tmar* (*Tmar* isolates were from two exposed fish and one cohabitant), and two isolates from the *Tmar* exposure were *Tdic* (both were from exposed fish). The remaining 11 isolates from the *Tmar* exposure were negative for all qPCR assays and could represent other non-target *Tenacibaculum* species, false-negatives, or bacteria with similar colony morphologies (e.g., *Cellulophaga* and *Dokdonia* spp.).

### 2.2. Clinical Signs of Infection

**Figure 1:**
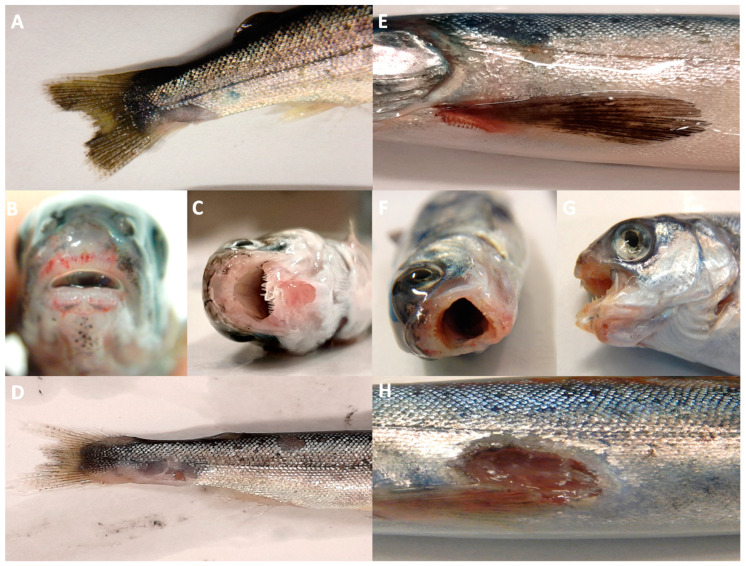
Clinical signs of mouthrot (tenacibaculosis) in Atlantic salmon (*Salmo salar* L.). (**A**–**D**) are fish exposed to *T. dicentrarchi* TdChD04, (**A**,**B**) were taken on day-1 (d 1) post-exposure, (**C**,**D**) were taken on d 2 post-exposure. (**E**–**H**) are fish exposed to *T. maritimum* NLF-15, (**E**,**F**) were taken on d 9–10 post-exposure, (**G**,**H**) were taken on d 17–18 post-exposure. Ulcerations and hemorrhages are present, predominately around the jaws (**B**,**C**,**F**,**G**), but also on the flanks and fins of fish (**A**,**D**,**E**,**H**). Yellow plaques were also visualized on the jaws of fish from the *T. maritimum* exposure (**F**,**G**). (**C**,**D**,**G**,**H**) display an exaggerated form of mouthrot, where the dentary bone and Meckel’s cartilage are broken, and surrounding tissues have been degraded (**C**,**G**); and ulcerations on the flank went beyond the epidermis into the musculature (**D**,**H**).

For the *Tdic* and *Tmar* treatment, deceased and moribund exposed and cohabitant fish presented with multifocal to regionally extensive ulcerations, predominately around the mouth, but also on the fins and flank of the fish (Figure 1). Yellow bacterial plaques were also identified on sections of the jaws and gills (Figure 1). Clinical signs of mouthrot (tenacibaculosis) became more severe over time corresponding to the occurrence of morbidity and mortality (Figure 1). Fish from *Tfinn* exposure experiments did not develop observable clinical signs of tenacibaculosis, or notable mortality (≥90% survival), and target bacteria could not be isolated from moribund or dead fish.

### 2.3. Kaplan–Meier Analysis

**Figure 2:**
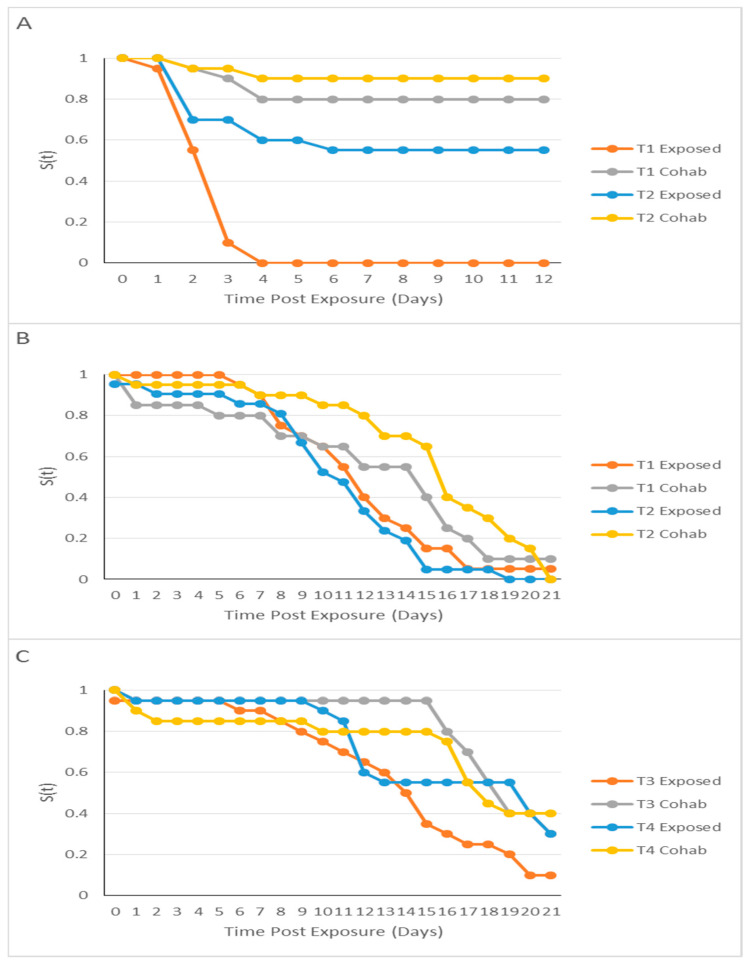
Kaplan–Meier Analysis of Atlantic salmon (*Salmo salar*) exposed to *Tenacibaculum* spp. treatments. Describing the probability of surviving (S[t]) over time. (**A**) is the *T. dicentrarchi* TdChD04 exposure for tank 1 (T1) and tank 2 (T2). (**B**) is the *T. maritimum* NLF-15 high concentration treatment for tank 1 (T1) and tank 2 (T2). (**C**) is the *T. maritimum* NLF-15 low concentration treatment for tank 3 (T3) and tank 4 (T4).

In Room 1, for the *Tdic* treatment (tank 1 [T1]–tank 2 [T2]), for T1, 0% (exposed) and 80% (cohabitant) survived to day 12 (d 12) (Figure 2A). For T2, 55% (exposed) and 90% (cohabitant) survived to d 12 (Figure 2A). For the *Tfinn* treatment (tank 3 [T3]–tank 4 [T4]), 90% or over survived for both groups until d 12 (data not shown). All controls for both rooms (tank 5 [T5]) did not experience mortality.

**Figure 3:**
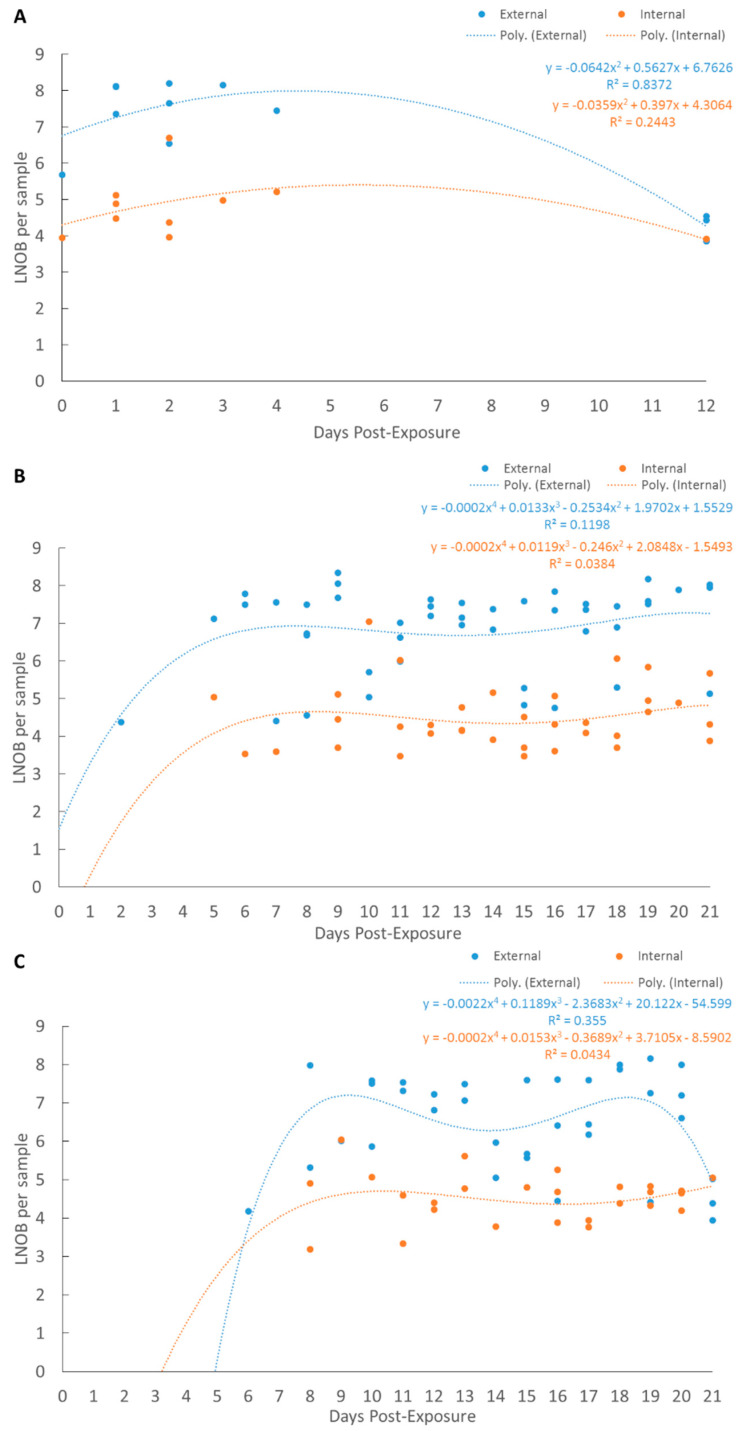
Regression models for the Log-number of bacteria (LNOB) recorded in external and internal tissues over time using *T. dicentrarchi* or *T. maritimum* specific qPCR assays. (**A**) is for the *T. dicentrarchi* treatment, (**B**) is for the *T. maritimum* high concentration treatment, (**C**) is for the *T. maritimum* low concentration treatment.

In Room 2, for the *Tmar* high concentration treatment (T1–T2), for T1, 5% (exposed) and 10% (cohabitant) survived to d 21 (Figure 3B). For T2, 0% for both groups survived to d 21 (Figure 2B). For the *Tmar* low concentration treatment (T3–T4), for T3, 10% (exposed) and 40% (cohabitant) survived to d 21 (Figure 2C). For T4, 30% (exposed) and 40% (cohabitant) survived to d 21 (Figure 2C).

### 2.4. qPCR Regression Modeling

*Tmar* or *Tdic* could not be identified in fish prior to the exposure. For each assay, there were significant differences between the Log-number of bacteria (LNOB) in internal and external tissues (*p* < 0.05), while there were no differences when comparing the LNOB recorded in exposed and cohabitant fish (*p* > 0.05) (Table 1). As a result, the data for each tank was subset based on the assay and internal or external tissues, but not based on exposed or cohabitant.

A quadratic regression model best described the LNOB throughout an infection for both external and internal tissues for the *Tdic* exposure (Figure 3A). From day 0 (d 0) until d 5, the LNOB increased 4–8 Log-units (Figure 3A). At the final sampling period, seven days after the last mortality, bacteria were still identified, but with either a 1–2 Log-unit decrease (external) or no change (internal) compared to throughout an infection (d 0–5 post-exposure) (Figure 3A). No collected or tested samples were positive for *Tfinn*; however, the few collected deceased fish from the *Tfinn* treatment were all positive for *Tdic*.

A quaternary model was thought to best describe the LNOB throughout an infection for both external and internal tissues for either *Tmar* exposure (Figure 3B,C). For the high concentration *Tmar* exposure after d 5, the LNOB bacteria increased 3–8 Log-units (Figure 3B). Around d 11–15 post-exposure, the trend-line decreases marginally, followed by a marginal increase until the end of the trial (Figure 3B). Similar patterns in the LNOB occurred for the low concentration *Tmar* treatment, but the main difference was that major increases in the LNOB do not begin to increase until around d 8 (Figure 3C).

### 2.5. Gross and Histopathology

**Figure 4:**
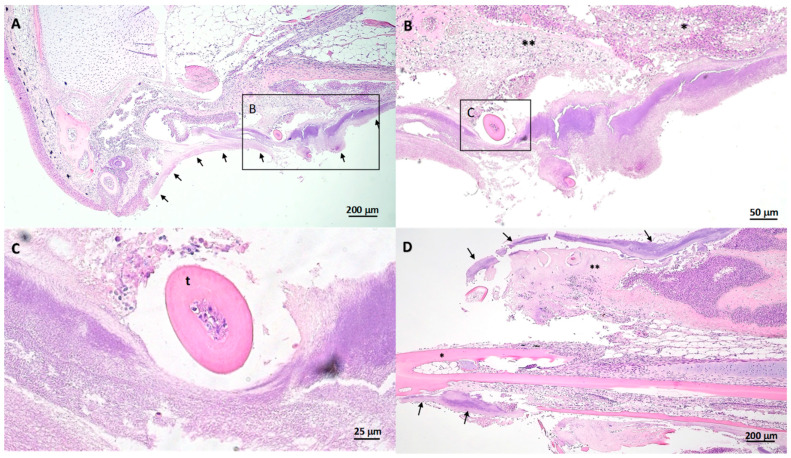
Histopathology of oral lesions from an Atlantic salmon smolt bath exposed to *T. dicentrarchi*. (**A**) Hematoxylin and eosin-stained sagittal section of the upper jaw with thick mats of thin filamentous bacteria (arrows) replacing areas of mucosa of the vomer and surrounding teeth. Black boxes in A and B indicate higher power magnification viewed in the next chronological alphabetical value. (**B**) Deep vomer ulceration overlying spongiotic epithelium (*) and necrotic submucosa (**). (**C**) Myriad long filamentous bacteria surrounding and replacing the submucosa around a tooth (t) of the vomer. (**D**) Oblique section of the lower jaw with fully exposed bone (*) with long filamentous bacterial mats (arrows) covering, replacing, and penetrating the oral mucosa and underlying viable and necrotic submucosa (**). Mats of similar bacteria (arrows) can be seen lining exposed bone and replacing dermal tissue on the underside of the jaw.

**Figure 5:**
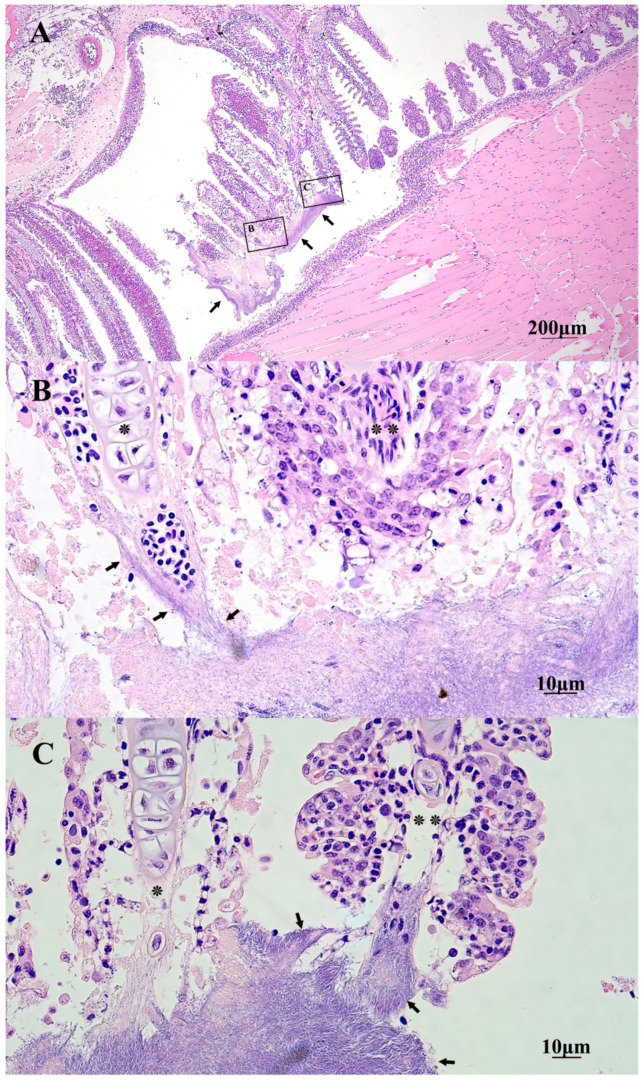
Histopathology of gill lesions from an Atlantic salmon smolt bath exposed to *T. maritimum*. (**A**) Hematoxylin and eosin-stained oblique section of the head and gills with necrotic and viable gill filaments covered by mats of long filamentous bacteria (arrows). Black boxes indicate higher power magnification as seen in (**B**,**C**). (**B**,**C**) Long filamentous bacteria (arrows) surrounding and penetrating blood vessels of a necrotic (*) and viable (**) gill filament, respectively.

**Figure 6:**
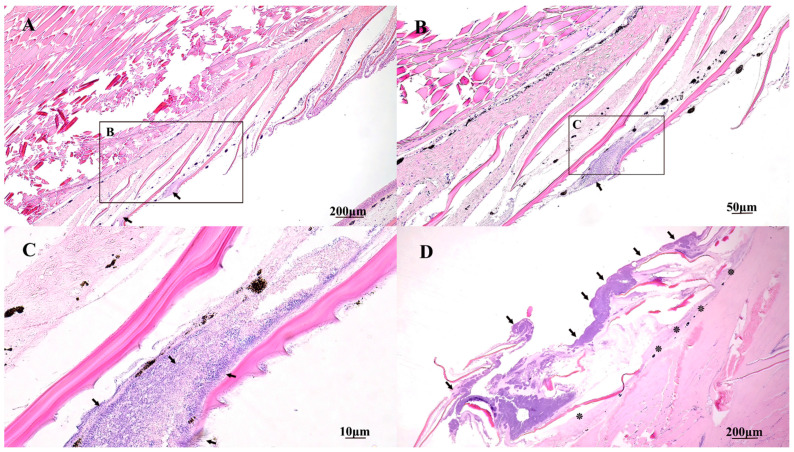
Histopathology of an Atlantic salmon smolt bath exposed to *T. maritimum*. (**A**) Hematoxylin and eosin-stained oblique section of the caudal trunk of the fish with regionally extensive epidermal erosion, ulceration, and intralesional bacteria (arrows). Scale pocket edema is also present adjacent to the ulcer. Black boxes in A and B surround intralesional bacteria and outline the areas of higher magnification as seen in (**B**,**C**). (**B**) Epidermis overlying scales is absent and bacterial mats (arrows) can be seen under scales. (**C**) Filamentous bacteria (arrows) replacing submucosa between scales and complete loss of epithelium on the external scale surface. (**D**) Large, deep epidermal ulceration of the lateral trunk of the fish with thick mats of intralesional bacteria (arrows) covering and penetrating (*) exposed dermis and underlying musculature.

The exposure to *Tmar* and *Tdic* induced similar gross and histological pathologies in moribund or deceased fish following direct exposure and cohabitation. Focal to widely extensive ulcerative lesions were present within and around the mouth (Figure 4), and occasionally on the body, tail, and gill (Figure 5 and Figure 6). Ulcerative lesions were often hemorrhagic, surrounded by the darkened dermis, and contained exposed bone (jaw or tail) or musculature (Figure 4). Notably, while the *Tfinn* exposure did not induce observable clinical signs of disease, the few mortalities in this group displayed a similar histological presentation to the *Tmar* and *Tdic* exposure groups. Mats of bacteria were grossly visible on gills from fish exposed to *Tmar*, but not on *Tdic* or *Tfinn* exposure or cohabitation fish (Figure 5) and few fish had grossly visible bacterial mats on the mouth, skin, and tail lesions. Lesion extent and severity did vary between individuals.

**Figure 7:**
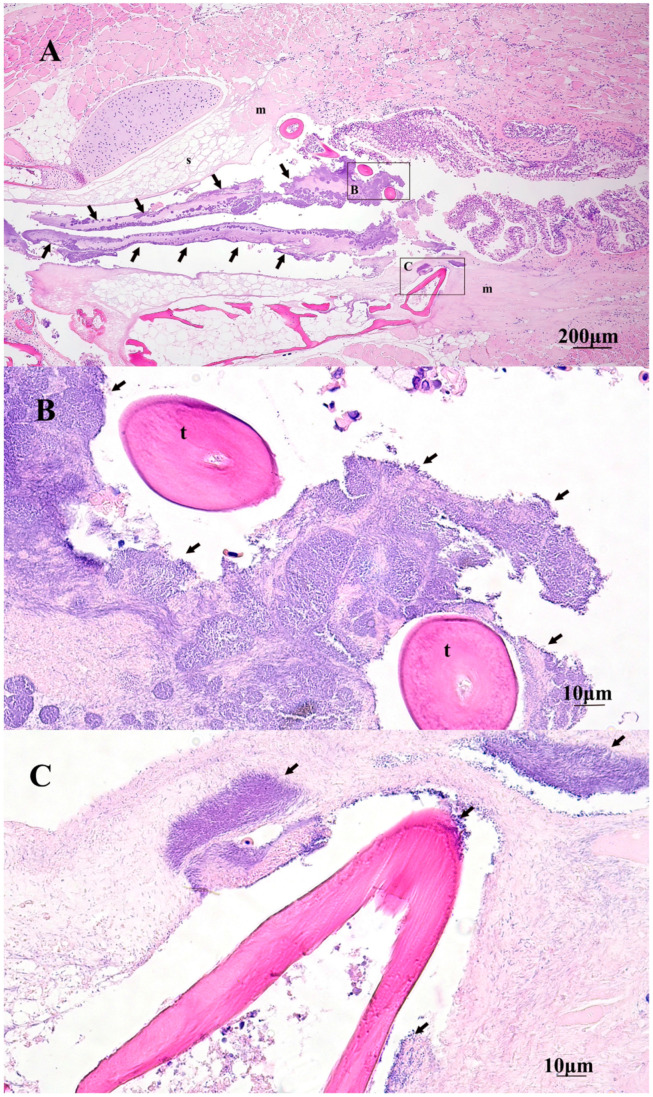
Histopathology of an Atlantic salmon smolt bath exposed to *T. maritimum*. (**A**) Hematoxylin and eosin-stained sagittal section of the buccopharyngeal cavity with extensive replacement of oral mucosa by thick mats of long filamentous bacteria (arrows). Sections of the submucosa (s) and deep muscle (m) bundles are undergoing degeneration and necrosis. Black boxes indicate higher power magnification as seen in (**B**,**C**). (**B**,**C**) Long filamentous bacteria (arrows) penetrating necrotic submucosa and surrounding buccopharyngeal teeth (t).

Histopathology of the oral lesions varied in severity and most cases were associated with mats of thin, long, rod-shaped, Gram-negative, bacteria similar to that of *Tmar*, *Tdic*, and *Tfinn* (Figure 4). External ulcerations were characterized by abrupt edges of intact epidermis above the upper and lower lips, and adjacent to the exposed hypodermis, deep dermis, bone, and or muscle. In some sections, mats of bacteria penetrated deep into the dermis and underlying musculature, lining exposed bony tissue (Figure 5). Little to no inflammation surrounded these lesions. When present, inflammatory populations were predominantly macrophages and lesser numbers of lymphocytes. Ulcerative lesions on the rostrum extended into the mouth and onto the vomer. Within the mouth, oral mucosa exhibited multifocal to regionally extensive spongiosis and hydropic degeneration with multifocal erosion, epidermal clefting and lifting, and loss of epidermis. Large and small mats of bacteria were present within ulcerative lesions and lined exposed bone of the vomer and also extended into gingival pockets. In some cases, mats of bacteria covered erosive and ulcerated oral mucosa near the oral esophageal junction (Figure 7). Similar to external lesions, small numbers of inflammatory cells surrounded bacteria that penetrated deep dermal tissues.

Few fish were observed with gross branchial lesions and low numbers of fish had multifocal to locally extensive mild to moderate hydropic generation and epithelial lifting on lamellae. Mats of long filamentous bacteria were observed in low numbers, which covered and penetrated normal and eroded sections of the epithelium lining the branchial arches and rakers (Figure 5).

Skin and tail lesions were observed in *Tdic* and *Tmar* exposure fish that had similar gross and histological presentations characterized by complete loss of epithelium and ulceration into the deep dermis and underlying musculature (lateral flank) or bone (tail). Mats of thin long bacteria replaced the epithelium and penetrated the deep dermis and caused multifocal degeneration of underlying muscle bundles (Figure 6). Scale pocket edema was present on multiple individuals; however, severity and extent of lesions were variable between fish. Similar to oral lesions, little to no inflammatory infiltrates were observed.

## 3. Discussion

Exposure to *Tmar* and *Tdic* resulted in mortality and similar clinical signs of mouth-rot. Unlike the *Tmar* treatments, there were large variations between survivorship for the *Tdic* treatment replicates for exposed fish. Inherit variations for survivorship have been recorded before for *Tenacibaculum*-related exposures; in a previous study, variation between replicates was at or below 20% [6]. Variations for survivorship, outside of the pathogen’s influence, may be related to host responses and the environment. The environment may be an important consideration, as variation could be related to the design of the RAS system, where tank-dependent effects may have occurred (e.g., differences in flow and aeration). However, with no major differences between survivorship for cohabitants between replicated, more research should occur to ensure that the variation between replicates for the survival of exposed and cohabitant fish is consistent. Interestingly, the *Tfinn* treatments did not result in noticeable mortality or clinical signs. The contrasting results are likely not species-dependent but isolate and dose-dependent, as *Tfinn* has been shown previously to induce tenacibaculosis in other experimental infection models [9]. The lack of clinical signs produced by *Tfinn* in this study could be influenced by genetic differences between isolates; the isolate of *Tfinn* used here may not have been virulent. This is likely because a selection of isolates were largely random and isolates collected from fish with lesions can contain both virulent and non-virulent isolates; for example, fish with coldwater disease [14]. The lack of demonstrated virulence may also be attributed to the methodology used, where bacteria may have not been in the correct conditions to produce lesions. Variable isolate pathogenicity has been described in other pathogens [15,16], including *Tenacibaculum* [6]. This could explain why *Tfinn* and even *Tmar* type-strain NCIMB 2154^T^ [6,9,17] have been related to mortality in fishes on-site, but not in-vivo, and could also explain why some isolates that are genetically identical using 16S rDNA have variable results in inducing disease in lab studies [6]. A third reason why *Tfinn* did not induce mouthrot was that it was outcompeted by a similar *Tenacibaculum* species for the same niche, as deceased fish for the *Tfinn* treatment were positive using qPCR for *Tdic*, but not *Tfinn*. Often when a new niche is available for colonization, bacteria that have similar resource requirements will compete, where three long-term consequences are often described (exclusion [a winner], assignment of metabolic niches, or spatial separation of bacteria) [18]. Contamination between tanks could have occurred, potentially through contaminated water, with *Tdic* as the winner and can be supported by the fact that *Tdic* is a common isolate from fish with mouthrot in BC [8]. This potential phenomenon requires further investigation, perhaps using dual infections and monitoring isolates over time with qPCR. 

Preliminary mathematical modeling of survival and bacterial enumeration in de-ceased fish may provide important insights to understanding mouthrot and tenacibaculosis. Prior research into aquatic pathogens has shown that cycle quotients from qPCR assays can differentiate disease states of fish and determine thresholds of bacteria required to cause infection, where similar applications have occurred for salmon gill poxvirus [19] and bacterial coldwater disease [20]. However, the qPCR assays developed by a previous study [7], which were used in the present study, are not suitable for determining such a threshold in uncontrolled conditions as there are limitations relating to the specificity and copy number for the 16S rDNA gene in *Tenacibaculum*. For *Tdic*, mortality of exposed fish within 48 h is similar to [4] and may indicate that the results of the *Tdic* exposure are not solely based on the bacteria. In this study, and work conducted previously [6], stress from the transition between freshwater and saltwater and exposure to bacteria could both play a significant role in the progression of disease in Atlantic salmon. This experimental model mimics the environmental challenges that salmon experience during the transition to netpen sites; in Canada, fish likely undergo considerable stress when directly transferred from freshwater hatcheries to marine sites. Shortly after the transition, mouthrot manifests and induces mortality in salmon until the fish reach a size threshold. Detection of *Tdic* in the present study was at low numbers after exposure and after mortality had ceased indicating that *Tenacibaculum* bacteria may become a commensal part of the microbiome as demonstrated for Atlantic salmon [10] and Asian sea bass (*Lates calcarifer*) [21]. However, without a greater understanding of the microbiome of Atlantic salmon, it is difficult to define what a ‘healthy microbiome’ is and understanding if that includes *Tenacibaculum*. In contrast, *Tmar* was detected at low numbers, which increased, fluctuated marginally, and then decreased. Despite these fluctuations, mortality and clinical signs of disease persisted until the end of the trial, with a low probability of survival for any group. *Tmar* may also induce disease in the host through exploiting dysbiotic changes to the microbiome; however, the ongoing or subacute disease profile, in contrast to the peracute one of *Tdic*, strongly suggests marked differences in pathogenesis between *Tenacibaculum* isolates. Additional complete genome sequencing, annotations, and comparisons would help identify why there could be differences in pathogenicity.

The pathogenesis of mouthrot could not be easily compared in this study, as fish were removed when moribund or dead, rather than sampling at a routine frequency throughout the trial similar to a previous experimental setup [22]. The current sampling protocol can show what the fish looked like at or near death but provides limited insights into the development of disease. Subsequent studies will use predetermined routine sampling to investigate the pathogenesis of tenacibaculosis. Even though the sampling routine was sub-optimal, important key ideas about the pathogenesis of tenacibaculosis were identified, such that from a bath exposure trial, *Tdic* and *Tmar* managed to spread internally. Further, this occurred for both the exposed fish and cohabitants indicating that tenacibaculosis is horizontally transmissible and can become systemic. Important key ideas are supportive of previous research [6], with a need for a greater understanding of the mechanisms for clinical sign presentation and mortality. Previous bioinformatics analysis of *T. maritimum* identified numerous genes and potential virulence factors related to secretion and uptake systems, mobility, adherence, and biosynthesis of compounds (i.e., hemolysins, proteases, glycoside hydrolases) [23]. These genes and resultant proteins require investigation to understand the pathogenesis of tenacibaculosis. In addition, complete genome comparisons between *Tenacibaculum* species would reveal if these same genes and protein targets can be used to develop criteria to identify which isolates may induce disease.

Even though other experimental trials have exposed fish to *Tenacibaculum* isolates [4,6,9], the described model is important because it uses isolates collected from BC waters and can provide important contrasts to other isolates inside [6] and outside of Canada [4,9]. The current research also demonstrated that several *Tenacibaculum* species can induce disease with the tested methodology.

## 4. Materials and Methods

### 4.1. Fish Husbandry

Atlantic salmon smolts (N = 400, 30 g) were raised in partial saltwater (11‰) by Grieg Seafood BC LTD. Prior to transport to the Centre for Innovation in Fish Health (CIFH), at Vancouver Island University in Nanaimo (BC), the salmon population was screened for and tested negative for infectious salmon anemia virus, infectious pancreatic necrosis virus, and piscine orthoreovirus. Water from the recirculating aquaculture systems (RAS) systems at the CIFH, were tested using qPCR for *Tmar*, *Tdic,* and *Tfinn* prior to the introduction of fish following previously developed protocols [7]. 

Following arrival at CIFH, fish were transferred from 11‰ saltwater to 30‰ saltwater in a RAS (~1200 L) containing five circular tanks (Tank [T] 1-T5; 100 L each, n = 40 per tank) in parallel, in two identical, but separate temperature- and humidity-controlled rooms (Room 1, 2) for 24 h. Water quality (temperature [12 °C], dissolved oxygen [~105% sat], pH [~8.0], oxidative-reductive potential [~350 mV], salinity [~30‰], flow rate [gal per hour]) was measured every 10 min using a real-time water quality monitoring system (Neptune Apex Controller System, Morgan Hill, CA, USA). Water from each tank was collected in the sump and treated by ultraviolet exposure (7–12 mW*cm^−2^) to reduce bacterial contamination before going back to the tanks. Fish were fed pellets (Nutra 1.5 mm, Skretting Vancouver, Vancouver, BC, Canada) twice daily (0.5% average body-mass). 

Humane endpoints were used and fish exhibiting clinical signs of illness (excessive ulcerative lesions, increased respiratory rates, loss of equilibrium, exophthalmia) at any point were euthanized using an overdose of tricaine methanesulfonate (Aqualife, 250 mg L^−1^, DIN 02168510, Syndel Canada, Nanaimo, BC, Canada) and counted as mortalities. To facilitate this, a high frequency of observation was used: pre-exposure, fish were checked three times a d; post-exposure, fish were checked six times a d for seven d, and four checks each d thereafter.

### 4.2. Bacterial Propagation

*Tmar* 2.1C, *Tdic* 20-4116-9, and *Tfinn* 20-4106-2 isolates used in this study were collected during 2019–2020 from Atlantic salmon during mouthrot outbreaks in BC waters. The three isolates were sequenced at the University of Alberta for the 16S rDNA sequence using the universal 27F and 1492R primers. Sequencing indicated that the three bacteria were most similar to *T. maritimum* NLF-15, *T. dicentrarchi* TdChD04, and *T. finnmarkense* Tsp.2; all three bacteria were compared using approximately 1400 bp and had percent identities above 98.6%. The three different *Tenacibaculum* species were used in separate exposures to compare mortality rates, clinical signs, and pathology. 

Isolates (*Tmar*, *Tdic*, and *Tfinn* [preserved in 1.4 ml aliquots (25% glycerol) at −80 °C]) were cultured on *Flexibacter maritimus* medium (FMM) supplemented with kanamycin (FMM+K) (50 µg mL^−1^) at 12 °C; downstream FMM+K broth cultures were mixed at 200 rpm. Standard curves were developed for each isolate using absorbances at 600 nm and colony-forming units (CFU) per mL of FMM+K broth (data not shown). For *Tdic* and *Tfinn*, 1 L of the medium, at an absorbance of 600 nm of 0.5 (2.0 × 10^9^ CFU*mL^−1^), was mixed with seawater (80 L) to a final concentration of 2.5 × 10^7^ CFU*mL^−1^. For *Tmar*, 100 mL of medium at an absorbance at 600 nm of 0.05 and 1.5 (2.0 × 10^9^ CFU*mL^−1^; 2.0 × 10^11^ CFU*mL^−1^) was cultured and mixed with seawater (80 L) to a final concentration of 2.5 × 10^6^ and 2.5 × 10^8^ CFU*mL^−1^.

### 4.3. Direct and Cohabitation Bath Exposures 

Three separate bath exposures on Atlantic salmon smolts were conducted simultaneously to determine clinical and pathogenic differences between *Tenacibaculum* species. Room 1 was used for *Tdic* and *Tfinn* exposures while Room 2 was used *Tmar* exposures (Table 2). For bath exposures, each room contained three, separate, static, aerated, 120 L exposure-tanks (ET) filled with 80 L of saltwater. Two ETs (ET1 & ET2) were used for bacterial exposures while ET3 was used as control exposure and contained an equivalent amount of sterile medium to the other ETs. Forty fish were placed in each 120 L ET at a stocking density of 12 kg m^−3^ and FMM broth (with and without bacteria) was added to obtain the final concentrations as described in Table 2. All ETs were covered, and fish were left for 5 h with aeration. Dissolved oxygen was measured every 30 min during the exposure (data not shown). After 5 h, 40 fish from each bacterial ET tank were split evenly into two 100 L RAS tanks (tank 1 [T1] & tank 2 [T2] or tank 3 [T3] & tank 4 [T4]) and were grouped with 20 naïve non-exposed cohabitants identified by adipose fin clipping (Table 2). The 40 fish from ET3 were put directly into RAS tank 5 [T5] with no cohabitant fish (Table 2). The experiment continued until there were no mortalities for 5 consecutive d and lasted for 21 d total

### 4.4. Pre- and Post-Exposure Sampling

Three pre-exposure fish from each room were sampled after the 24 h acclimation, euthanized as described, grossly examined, and swabbed from the mouths, gills, and head-kidneys for bacterial culture on FMM+K. Bacterial plates were incubated at 12 °C for a minimum of 7 d and colony morphologies were recorded. From each fish, external (jaw and gills), and internal (spleen and head-kidney) tissues were collected into RNAlater (Ambion Inc., SIGMA: R0901-500ML, Austin, TX, USA), cooled overnight at 4 °C before being stored at −80 °C. Sagittal sections of the fish, such as head, body, and internal organs were placed into 10% neutral buffered formalin (NBF) for histopathology.

Post-exposure, deceased and or moribund fish were removed and processed similar to that of pre-exposure fish. At each time check, a maximum of three deceased or moribund fish from each tank was processed for downstream testing. For each fish, gross lesions were identified and recorded prior to necropsy examination and retrieval of tissues for downstream qPCR and histopathology. At the end of the trial, three fish from each tank (if remaining) were euthanized as described and processed similar to pre-exposure fish.

### 4.5. Quantitative PCR

DNA from collected tissues and bacterial isolates were extracted using the OMEGA E.Z.N.A Tissue DNA extraction kit (Omega Bio-tek, Inc., Norcross, GA, USA) according to the manufacturer’s recommendations, with the following modification: fish tissues were incubated at 56 °C in lysis buffer for 24 h before extraction.

Three qPCR assays specific to *Tmar* [24,25], *Tdic,* and *Tfinn* [7] were used on tissue samples and bacterial isolates. The procedure and thermal profiles designed previously [7,24] were used with minor modifications: the *Tmar* probe (0.05 μM) the *Tmar* primers (0.5 μM); and the *Tdic* probe (0.125 μM), per reaction. Primers were obtained from SIGMA-ALDRICH (Canada) and probes were obtained from Eurofins Genomics (USA). For each reaction, each well received 100 ng of sample DNA. Similar to a previous study [7], a cycle quotient of 35 was used as a cut-off to represent no bacteria.

Standard curves, using spiked muscle and kidney tissues (100 ng of salmon tissue DNA and 0.001–100 ng of bacterial DNA) were used for each assay to ensure proper amplification efficiency (data not shown). With no differences between the standard curves based on tissue type (data not shown), an average of both standard curves was applied. Three equations based on generated standard curves (one for the *Tmar* specific assay, a second for the *Tdic* specific assay, and a third for the *Tfinn* specific assay) were used to change the qPCR cycle-quotient output to a theoretical number of bacteria per g of fish tissue (Table 2). The equations stem from a previous study [7]. For each equation, ‘x’ is the Log-number of bacteria (LNOB) per gram of fish tissue, ‘c’ is the concentration of the extracted sample and ‘v’ is the volume of the extracted sample (Table 3).

### 4.6. Histopathology

Tissue processing, sectioning (7 µm) of paraffin-embedded tissue, and hematoxylin and eosin staining were performed by a commercial laboratory (Animal Health Laboratory, Abbotsford, BC, Canada). Each cassette contained a sagittal section of the head and any additional lesions to the body such as the tail.

### 4.7. Statistics

Statistical analysis was performed on R-Studio version 3.6.1 [26]. Normal distributions and homogenous variances were tested using Q-Q plots, the Shapiro-Wilcoxon test, and the Levene test. Kaplan–Meier analyses were conducted using the R package ‘*survival*’ for each tank population (exposed or cohabitant) using the daily mortality data. Kruskal–Wallis tests for the qPCR results, were conducted between: assays, cohabitants, and exposed fish, and internal and external tissues to subset the data for regression modeling and subsequent comparison by ANOVA.

## 5. Conclusions

Mouthrot was successfully induced in Atlantic salmon using multiple *Tenacibaculum* species; where dysbiosis [10,11] through a large stress event (i.e., transfer from freshwater to saltwater [6]) may be required for mouthrot to manifest in BC Atlantic salmon smolts. Further investigation of genetic differences comparing whole genomes among *Tenacibaculum* species, strains, and isolates is needed to identify potential differences in virulence mechanisms that result in variation in clinical signs. Similarly, investigations of the relationship between stress and dysbiosis, and how and why mouthrot occurs in Atlantic salmon are needed to understand disease pathogenesis to define future aquaculture management practices to prevent this costly disease.

## Figures and Tables

**Table 1:** Kruskal–Wallis test output to determine how to subset the Log-number of bacteria (LNOB) for regression modeling. The assay is either specific to *T. dicentrarchi* (*Tdic*) or *T. maritimum* (*Tmar*).

Room	RAS Tank	Specific to	Comparison *	Mean LNOB ± SD	KW ^α^	df	*p*
1	1 & 2	*Tdic*	Int. and Ext. tissues	Int: 4.66 ± 0.909 Ext: 5.96 ± 1.69	4.5	1	0.034
2	1–4	*Tmar*	Int. and Ext. tissues	Int: 4.51 ± 0.758 Ext: 6.33 ± 1.39	72	1	<2.2 × 10^−16^
1	1 & 2	*Tdic*	E and C fish	E: 5.49 ± 1.58 C: 5.80 ± 1.89	0.66	1	0.41
2	1–4	*Tmar*	E and C fish	E: 5.71 ± 1.48 C: 5.75 ± 1.52	0.021	1	0.88

*: Internal (Int) tissues, external (Ext) tissues, exposed (E), and cohabitant (C) fish. α: Kruskal–Wallis chi-squared value (KW).

**Table 2:** *Tenacibaculum* bath-exposure information. Room 1 was used for *T. dicentrarchi and T. finnmarkense* exposure trials and Room 2 for *T. maritimum* exposure trials. For each room, each exposure tank (ET) number paired with the treatment, final concentration (Colony-forming units [CFU]*mL^−1^) of the treatment, number of exposed fish, duration of exposure (hr), location of fish post-exposure, and the number of fish in each recirculating aquaculture system (RAS) tanks (T) are described.

Room	ET#:Treatment	Final Concentration	Number Exposed	Duration of Exposure (hr)	Post-exposure RAS Tank (T) Location	Number in Each RAS Tank *
1	ET1: *T. dicentrarchi* TdChD04	2.5 × 10^7^	40	5	T1 & T2	20E & 20C
1	ET2: *T. finnmarkense* Tsp.2	2.5 × 10^7^	40	5	T3 & T4	20E & 20C
1	ET3: Sham-Control (medium only)	0	40	5	T5	40E
2	ET1: *T. maritimum* NLF-15	2.5 × 10^6^	41	5	T1 & T2	20/21E ^α^ & 20C
2	ET2: *T. maritimum* NLF-15	2.5 × 10^8^	40	5	T3 & T4	20E & 20C
2	ET3: Sham-Control (medium only)	0	40	5	T5	40E

* E = Exposed, C = Cohab α = 20 exposed fish in tank 1, 21 in tank 2.

**Table 3:** Equations used to convert cycle-quotient values from qPCR to the Log-number of *Tenacibaculum* spp. bacteria per gram of fish tissue. For each equation, ‘x’ is the Log-number of bacteria (LNOB) per gram of fish tissue, ‘c’ is the concentration of the extracted sample and ‘v’ is the volume of the extracted sample.

Specific to	Assay Equation	Reference for Assay
*T. maritimum*	x = LOG((((10^(Mean Cq−34.4)/−3.2))^/10)/100)*c*v)*33.3) *	[24]
*T. dicentrarchi*	x = LOG((((10^(Mean Cq−36.9)/−3.4))^/10)/100)*c*v)*33.3)	[7,25]
*T. finnmarkense*	x = LOG((((10^(Mean Cq−38.4)/−3.6))^/10)/100)*c*v)*33.3)	[7,25]

* Indicates that the equation originated from [25].

## Data Availability

Data can be accessed through the corresponding author (Joseph Nowlan) or the principal investigator (Spencer Russell).
